# Ticagrelor, but Not Clopidogrel, Attenuates Hepatic Steatosis in a Model of Metabolic Dysfunction-Associated Steatotic Liver Disease

**DOI:** 10.3390/nu16070920

**Published:** 2024-03-22

**Authors:** Eun Jeoung Lee, Seung Min Lee, Ju Hee Oh, Hye Young Kim, Waqar Khalid Saeed, Hyun Sung Kim, Dae Won Jun

**Affiliations:** 1 Department of Translational Medicine, Graduate School of Biomedical Science and Engineering, Hanyang University, Seoul 04763, Republic of Korea; cek940518@naver.com (E.J.L.); tmdals0323@hanyang.ac.kr (S.M.L.); kkimhy0927@hanyang.ac.kr (H.Y.K.); 2 Department of Obstetrics and Gynecology, Institute of Women’s Medical Life Science, Yonsei Cancer Center, Severance Hospital, Yonsei University College of Medicine, Seoul 03722, Republic of Korea; onk1210@yuhs.ac; 3 Department of Biomedical Sciences, Pak-Austria Fachhochschule—Institute of Applied Sciences and Technology, Mang 22621, Pakistan; waqar.saeed33@gmail.com; 4 Department of Pathology, Hanyang University School of Medicine, Seoul 04763, Republic of Korea; hhnt5841@gmail.com; 5 Department of Internal Medicine, Hanyang University School of Medicine, Seoul 04763, Republic of Korea

**Keywords:** non-alcoholic fatty liver disease, liver biopsy, clopidogrel, ticagrelor, lipogenesis

## Abstract

Background: Previous studies have suggested that platelets are associated with inflammation and steatosis and may play an important role in liver health. Therefore, we evaluated whether antiplatelet agents can improve metabolic disorder-related fatty liver disease (MASLD). Methods: The mice used in the study were fed a high-fat-diet (HFD) and were stratified through liver biopsy at 18 weeks. A total of 22 mice with NAFLD activity scores (NAS) ≥ 4 were randomly divided into three groups (HFD-only, clopidogrel (CLO; 35 mg/kg/day), ticagrelor (TIC; 40 mg/kg/day) group). And then, they were fed a feed mixed with the respective drug for 15 weeks. Blood and tissue samples were collected and used in the study. Results: The TIC group showed a significantly lower degree of NAS and steatosis than the HFD group (*p* = 0.0047), but no effect on the CLO group was observed. Hepatic lipogenesis markers’ (SREBP1c, FAS, SCD1, and DGAT2) expression and endoplasmic reticulum (ER) stress markers (CHOP, Xbp1, and GRP78) only reduced significantly in the TIC treatment group. Inflammation genes (MCP1 and TNF-α) also decreased significantly in the TIC group, but not in the CLO group. Nile red staining intensity and hepatic lipogenesis markers were reduced significantly in HepG2 cells following TIC treatment. Conclusion: Ticagrelor attenuated NAS and hepatic steatosis in a MASLD mice model by attenuating lipogenesis and inflammation, but not in the CLO group.

## 1. Introduction

Metabolic dysfunction-associated steatotic liver disease (MASLD) is the most common chronic liver disease worldwide, and its prevalence has been increasing significantly in Asia [1]. Moreover, MASLD is becoming a leading cause of hepatocellular carcinoma and liver-related deaths [1]. MASLD is a multifactorial disease: obesity, insulin resistance, and metabolic dysfunction can all affect its pathogenesis [2,3].

In recent years, studies have found that platelets play a key role in the inflammatory and immune responses to MASLD [3]. For instance, Malehmir et al. demonstrated that, when fatty liver occurs, platelets enter the liver and bind to GPlbα on the surface of other platelets, leading to inflammation. In addition, both inflammation and the number of immune cells are reduced when patients are administered an antibody that blocks platelet glycoproteins [4]. Previous studies have also reported that antiplatelet therapy attenuates the progression of MASLD to non-alcoholic steatohepatitis [3]. Another study suggested that antiplatelet drugs, including acetylsalicylic acid and P2Y12 receptor antagonists, protect against liver fibrosis [5], while Iannacone et al. proposed that aspirin or clopidogrel (CLO)-induced reductions in platelet activation and aggregation reduce tumor formation and liver damage [6]. Likewise, animal studies have shown that another antiplatelet drug, cilostazol, also reduces liver steatosis and inflammation [7]. In addition to their role in coagulation, platelets release pro-inflammatory mediators and interact with other cells. Both ticagrelor (TIC) and CLO reduce the expression of inflammatory cytokines [8].

TIC and CLO are the most widely used P2Y12 receptor antagonists worldwide. The effects of TIC are reversible and involve direct binding to the P2Y12 receptor. In contrast, CLO binds irreversibly to P2Y12, is converted to an active metabolite through liver metabolism, and inhibits ADP signaling [9]. Though both TIC and CLO target the P2Y12 receptor, TIC showed different results in terms of effectiveness and side effects such as bleeding [10].

Recently, the role of platelets in liver diseases has been emphasized, particularly in the context of fatty liver disease. Platelets can bind to human hepatic endothelial cells, triggering the release of CXCL8 and CCL2, which in turn attract immune cells and potentially contribute to liver damage. Although widely used drugs like clopidogrel and aspirin face challenges in reversing fatty liver disease, these research findings suggest potential therapeutic targets. When considering conditions such as cardiovascular diseases, stroke, and thrombosis, understanding the significance of antiplatelet agents in liver pathophysiology can guide treatment decisions. While ticagrelor currently faces limitations as a fatty liver therapy, this research sheds light on the importance of antiplatelet strategies, which may prove beneficial for patients with fatty liver.

In the present study, we examined the effects of TIC and CLO in an animal model of NAFLD. We also investigated the differences between the two drugs and the mechanisms of their effects.

## 2. Methods

### 2.1. Animal Experiments

**Figure 1:**
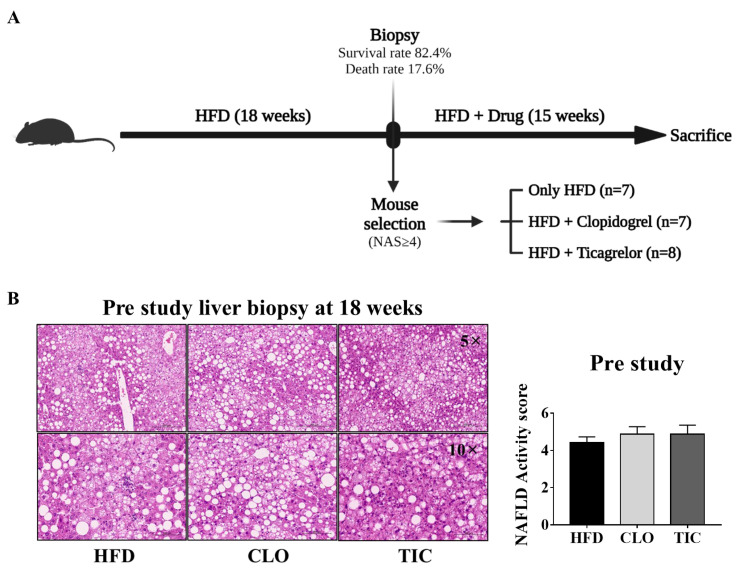
Experiment protocol and biopsy results. (**A**) Design of the animal experiment. (**B**) Hematoxylin and eosin staining result and NAFLD activity score at pre-biopsy. HFD, high-fat diet; CLO, clopidogrel; TIC, ticagrelor. Data are presented as mean ± SEM, analyzed using one-way ANOVA.

Six-week-old C57BL/6 mice (*n* = 34; Central Lab. Animal Inc., Seoul, Republic of Korea) were maintained at the Hanyang Laboratory Animal Research Center. The animals were kept in a pathogen-free facility with controlled temperature (23 °C ± 2 °C) and humidity (55% ± 5%). They followed a 12 h artificial light/dark cycle and were fed a high-fat diet (60%; D12492; Research Diets) for 33 weeks. A pre-study biopsy was performed to determine the NAFLD activity score (NAS) on the 18th week [11]. From the 18th week until the end of the study, body weight and food intake were measured weekly. Mice were euthanatized via an intraperitoneal injection of Zoletil (Virbac Laboratories, Carros, France) and Rompun (Bayer Korea, Seoul, Republic of Korea) on the 33rd week (Figure 1A), and liver tissue and serum samples were collected. The research received approval from the Hanyang University Institutional Animal Care and Use Committee (HY-IACUC-20-0033).

### 2.2. Biopsy-Proven MASLD Model Using Pre-Study Biopsy

In order to establish a metabolic dysfunction-associated steatotic liver disease (MASLD) model in animal models with fatty liver, liver tissue biopsies are performed. This is because a high-fat diet does not always induce fatty liver in animals, and the degree of fatty liver induction varies among different animals. Therefore, an MASLD model validated through tissue biopsies can help correct inter-individual differences in research outcomes and enhance the reliability of the results [11]. A biopsy-proven NAFLD model was created using a pre-study biopsy, according to previous methods [12]. On the 18th week, mice were anesthetized via an i.p. injection of Tiletamine (Zoletil; Virbac Laboratories, Carros, France) and Xylazine (Rompun; Bayer Korea, Seoul, Republic of Korea). The abdomens of the mice were shaved and disinfected using a 10% iodine solution. Each liver was accessed through an incision in the middle of the abdomen and taken out using surgical scissors. The length of each obtained liver was approximately 0.9 cm. After the biopsy, bleeding was stopped using a heated spatula, and the incision was sutured. After surgery, the animals were warmed using a heat lamp and given drinking water containing tetracycline for 3 days. The administration of aspirin or acetaminophen was considered for the relief of the animals’ pain. However, since there have been previous studies that showed that analgesics are effective in alleviating diseases in nonalcoholic fatty liver disease [13,14,15], this experiment was not used to confirm disease improvement through drug administration in liver disease animal models.

### 2.3. Randomization and Stratification of Animals

Hematoxylin and eosin-stained liver tissue sections were scored to calculate the NAFLD activity score (NAS). During this process, pathological tests were performed blindly, and mice with NAS scores < 4 were excluded from the study due to insufficient fatty liver induction. NAS was assessed using a previously established scoring system [16]. A total of 22 mice with NAS ≥ 4 were randomly divided into HFD (4.4 ± 0.3, *n* = 7), HFD with CLO (4.9 ± 0.4, *n* = 7), and HFD with TIC (4.9 ± 0.5, *n* = 8) groups. After randomization, the animals were fed an HFD and treated using either CLO (35 mg/kg/day) or TIC (40 mg/kg/day) for 15 weeks [17,18,19,20,21]. The drug was mixed with the feed given to the animals.

### 2.4. Cell Culture

HepG2 cells (American Type Culture Collection; ATCC, Manassas, VA, USA) were kept at 37 °C with 5% CO_2_ in low-glucose Dulbecco’s Modified Eagle’s Medium (DMEM; Gibco, Grand Island, NY, USA) with 10% fetal bovine serum (FBS; Gibco) and 1% penicillin and streptomycin (Gibco). The medium was changed to a new medium every 2 days. HepG2 cells were seeded (1 × 10^6^/well) in a six-well plate in DMEM. And after 24 h, the cells were treated with oleic acid (OA; 300 µM), CLO (10 µM), or TIC (10 µM). Cells were treated simultaneously with OA, TIC, and CLO for 24 h.

### 2.5. Biochemical Analysis

Blood was collected from the heart using an insulin syringe and stored in serum-separating tubes (BD Vacutainer SST Tube, 367989). The serum was separated by centrifugation at 3000 rpm for 15 min, and the separated serum was stored at −80 °C. Cholesterol, triglyceride, aspartate transaminase (AST), and alanine transaminase (ALT) were analyzed using a Hitachi 747 autoanalyzer (Hitachi, Tokyo, Japan) according to the manufacturer’s instructions (Knotus Co. Ltd., Incheon, Republic of Korea).

### 2.6. Histological Analysis

Mouse liver tissues were fixed in 4% paraformaldehyde (PFA) solution, embedded in paraffin, and sectioned (4 μm). Sections were used for hematoxylin and eosin (H&E) staining and Sirius red staining. To evaluate fibrosis, sections were stained using Picro Sirius red solution (Abcam, Cambridge, MA, USA) for 25 min. The stained tissue sections were captured using a slide scanner known as the Zeiss AxioScan (AxioSan.Z1; Zeiss, Oderkochen, Germany). An independent blinded researcher assessed all histological data.

### 2.7. RNA Sequencing Analysis

The RNA sequencing data from previous studies were analyzed. The processed data of the RNA sequencing of the liver tissues of mice treated using TIC or CLO were used on the ArrayExpress (https://www.ebi.ac.uk/biostudies/arrayexpress, accessed on 6 February 2024) server, with the accession number E-MTAB-8049 [20].

### 2.8. RNA Extraction and qRT-PCR Analysis

Total RNA was isolated from HepG2 cells and mouse livers using a TRIzol reagent (Invitrogen Co., Waltham, MA, USA). In total, 3 μg of extracted RNA was reverse transcribed into cDNA using reverse transcriptase (PrimeScript™ RT Reagent Kit; TaKaRa, Kusatsu, Japan). Quantitative real-time PCR (qRT-PCR) amplification was carried out on a LightCycler 480 (Roche Diagnostics, Indianapolis, IN, USA) with LightCycler 480 SYBR Green I Master mix (Roche Diagnostics). RNA expression levels were analyzed using the LightCycler^®^ program, and all experiments were conducted in triplicate. The measured values were normalized to the expression levels of GAPDH and β-actin. Primer sequences are provided in Table 1.

### 2.9. Protein Extraction and Western Blot

Proteins extracted from whole liver tissue were obtained using RIPA lysis buffer (GenDEPOT, Hanam, Republic of Korea). Twenty micrograms of these proteins was then loaded onto a 10% sodium dodecyl sulfate–polyacrylamide gel electrophoresis gel. Following electrophoresis, the proteins were transferred to a nitrocellulose (NC) membrane (pore size 0.45 μm, Bio-Rad, Hercules, CA, USA). The transferred membrane was incubated with 1X EzBlock Chemi solution (ATTO, Tokyo, Japan) for 30 min, followed by overnight incubation with primary antibodies at 4 °C. The primary antibodies used are listed in Table 2. Subsequently, the membrane was incubated with a secondary HRP-conjugated anti-mouse antibody (1:5000, Jackson Immunoresearch, West Grove, PA, USA) at room temperature for 1 h. β-Actin served as the protein loading control. The transferred membranes were visualized using the Dyne ECL STAR Western blotting detection kit (Dyne Bio, Seongnam, Republic of Korea), and the results were quantified using an image analyzer (Image Lab 3.0, Bio-Rad, Hercules, CA, USA). All of the primary antibodies are described in Table 2.

### 2.10. Nile Red Staining

HepG2 cells were seeded (5 × 10^6^/well) in a 24-well plate in DMEM and incubated for 24 h. The cells were treated with OA (100 µM), CLO (10 µM), or TIC (10 µM) for 24 h. The cells were treated with OA, CLO, or TIC simultaneously. The cells were then washed with phosphate-buffered saline (PBS), fixed with 4% PFA for 15 min, and stained with Nile red solution (1 μg/mL) in the dark for 30 min at room temperature. Next, the cells were washed and mounted using a DAPI-containing mounting solution (H-1200; Vector Laboratories, Newark, CA, USA). Nile red staining was visualized using a fluorescence microscope (Leica DMI4000B; Leica, Wetzlar, Germany), and fluorescence intensity was measured using ImageJ software 1.53a (NIH, National Institutes of Health).

### 2.11. Gas Chromatography–Mass Spectrometry 

HepG2 cells were seeded in six-well plates and incubated for 24 h. Palmitic acid (PA) (300 µM) and OA (100 µM) co-treated HepG2 cells were treated with either TIC (100 nM, 500 nM, 1 µM, 10 µM) or CLO (100 nM, 500 nM, 1 µM, 10 µM). After 24 h, the medium was removed and washed twice with PBS. For cell lysis, cold methanol (1.5 mL; 80/20 *v*/*v*) was added to each well, followed by vortexing for 1 min. Next, an internal standard solution was added (50 µL; 0.1 mg/mL, C14:0 Myristic acid-d27 [IS]), followed by centrifugation at 14,000 rpm for 10 min at 4 °C. Supernatants were collected, and 1.8 mL of water and 0.96 mL of chloroform were added to each sample. All cell samples were assessed using an Agilent 7890/5975 instrument (Agilent, Santa Clara, CA, USA), according to the manufacturer’s instructions (Asan Hospital Metabolomics Core Lab, Seoul, Republic of Korea).

### 2.12. Statistical Analyses

The Statistical Package for GraphPad Prism 7.0 (GraphPad Software, Inc., San Diego, CA, USA) was used for statistical analyses. All data are expressed as the mean ± standard error of the mean (SEM). Data were analyzed using one-way ANOVA analysis (for multiple comparisons), and post hoc multiple comparisons were made with Tukey’s test, which assumes equal variances. Statistical significance was set at a *p*-value < 0.05.

## 3. Results

### 3.1. Biopsy-Proven MASLD Model and Stratification Using Pre-Study Biopsy

All mice were fed an HFD for 33 weeks to establish the MASLD model (Figure 1A). On the 18th week, a pre-study liver biopsy was performed to assess NAS and allow mouse selection. A total of 34 mice were biopsied. The pre-study-related mortality rate was 17.6% (*n* = 6). Among the remaining 28 mice, 22 had an NAS ≥ 4 (mean: 4.7 ± 1.1) and were allocated to the three arms (HFD group, CLO, and TIC treatment group); the six mice with an NAS < 4 were excluded from the study. Biopsied liver tissue specimens were stained using hematoxylin and eosin to assess NAS (Figure 1B). The baseline NAS was similar among the three groups (HFD group: 4.4 ± 0.3, CLO group: 4.9 ± 0.4, TIC group: 4. 9 ± 0.5).

### 3.2. Ticagrelor Decreased Steatosis and Overall NAS

**Figure 2:**
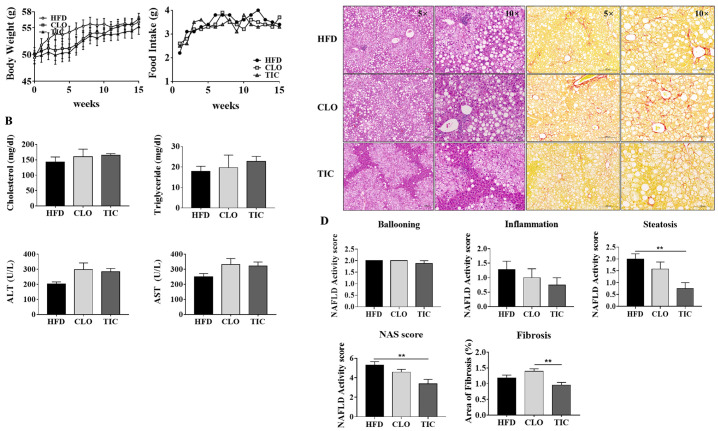
Changes in body weight, food intake, liver weight, biochemical markers, and histological differences in the animal experiment. (**A**) Body weight, food intake, and liver weight remained unchanged in all groups, as did (**B**) cholesterol, triglyceride, ALT, and AST levels. (**C**) Liver histology was examined through H&E staining (left, 50×) and Sirius red staining (right, 100×). (**D**) Comparison of steatosis, hepatocyte ballooning, lobular inflammation, NAFLD activity score, and area of fibrosis in the mouse liver specimens of each treatment group. The percentage of fibrosis area was quantified using Sirius red staining. Data are presented as mean ± SEM, analyzed using one-way ANOVA. ** *p* < 0.01 compared with HFD and CLO.

There were no differences among the three groups in terms of body weight (HFD group: 56.1 ± 2.5 g, CLO group: 56.7 ± 2.5 g, TIC group: 55.2 ± 3.7 g) or liver weight (HFD group: 3.1 ± 0.2 g, CLO group: 3.1 ± 0.4 g, TIC group: 2.5 ± 0.7 g) (Figure 2A). Food intake did not differ among the groups, and there were no differences in the serum alanine aminotransferase (ALT), aspartate aminotransferase (AST), cholesterol, or triglyceride levels between the TIC and CLO groups (Figure 2B). H&E staining was performed to evaluate the histological differences among the groups (Figure 2C). The degree of hepatic steatosis and total NAS were only lower in the TIC group compared to the HFD control group (Figure 2D). However, the NAS, degree of steatosis, and inflammation were not different between the HFD control and CLO treatment groups. In addition, the stage of hepatic fibrosis was lower in the TIC group than in the CLO group.

### 3.3. Ticagrelor Decreased De Novo Lipogenesis and ER Stress Markers

**Figure 3:**
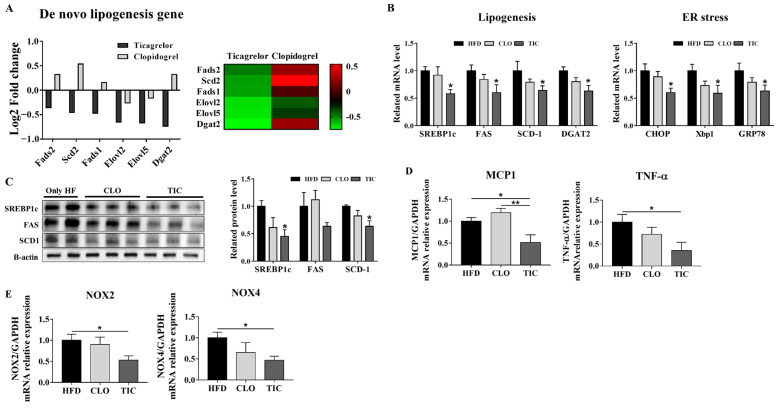
Expression of hepatic lipogenesis and inflammation-related genes in liver tissues. (**A**) De novo lipogenesis gene expression analysis from RNA sequencing data. (**B**) mRNA expression of lipogenesis and ER stress markers was evaluated in liver tissue after treatment. (**C**) Protein expression of lipogenesis markers was evaluated in liver tissues after treatment. (**D**,**E**) mRNA expression of inflammatory markers and reactive oxygen species production markers were evaluated in liver tissue. Data are presented as mean ± SEM, analyzed using one-way ANOVA. * *p* < 0.05 compared with HFD, ** *p* < 0.01 compared with CLO.

We compared hepatic mRNA transcriptome data related to de novo triglyceride synthesis between the CLO and TIC groups [21]. Based on public RNA sequencing data, the expression of de novo lipogenesis-related genes was lower in the TIC group than in the CLO group (Figure 3A). We confirmed that the expression of hepatic de novo lipogenesis markers was significantly lower in the TIC group than in the HFD group (Figure 3B). The protein expression of SREBP1c, FAS, and SCD-1 was also lower in the TIC group than in the HFD group (Figure 3C). The expression of ER stress markers (CHOP, Xbp1, and GRP78), inflammatory cytokines (MCP1 and TNF-α), and NOX 2/4 was also significantly lower in the TIC group than in the HFD group (Figure 3B,D).

### 3.4. Ticagrelor Decreased Intracellular Fat Deposition

**Figure 4:**
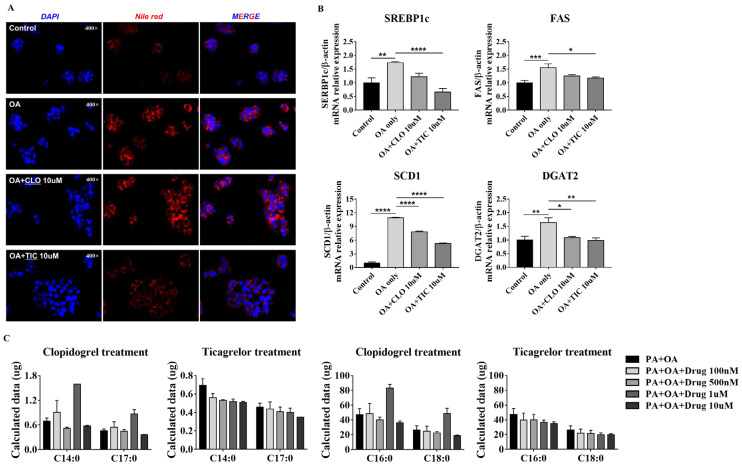
Nile red staining and expression of lipogenesis genes in HepG2 cells. (**A**) Nile red staining was conducted after induction with oleic acid (OA) for 24 h. (**B**) mRNA expression of lipogenesis markers was evaluated after induction using OA-only treatment or OA + drugs for 24 h. (**C**) HepG2 cells co-treated with palmitic acid (300 µM) and OA (100 µM) were treated using either clopidogrel or ticagrelor (100 nM, 500 nM, 1 µM, 10 µM) for 24 h. Data are presented as mean ± SEM, analyzed using one-way ANOVA. * *p* < 0.05, ** *p* < 0.01, *** *p* <0.001, **** *p* < 0.0001 compared with control and OA-only group.

Next, Nile red staining was performed to quantify the intracellular lipid content. TIC (10 µM) treatment decreased Nile red fluorescence by 2.5-fold in HepG2 cells compared with OA treatment alone (Figure 4A). Consistent with the Nile red staining results, TIC (10 µM) treatment also significantly decreased SREBP1c, FAS, SCD1, and DGAT2 expression (Figure 4B). We performed gas chromatography–mass spectrometry to determine changes in lipid composition. TIC treatment decreased C14:0, C17:0, C16:0, and C18:0 in a concentration-dependent manner in PA and OA co-treated HepG2 cells, though the difference was not significant (Figure 4C).

## 4. Discussion

Our study showed that TIC significantly reduced steatosis and NAS in an animal model of MASLD. TIC also decreased the expression of de novo lipogenesis genes (SREBP1c, FAS, SCD1, and DGAT2) in the MASLD model and in OA-treated HepG2 cells. Moreover, TIC, but not CLO, reduced the expression of hepatic inflammatory markers, including TNF-α and MCP1. In contrast, CLO did not improve steatosis nor inflammation. 

The strength of the present study was that we used a biopsy-proven MASLD model as well as stratification according to MASLD severity in mice. We performed the animal study that was most similar to a human clinical trial [11,22]. There is significant variation in the HFD-induced MASLD animal study [12], and we attempted to overcome the shortcomings of pre-clinical studies using pre-study liver biopsies and stratification. The NAS score was determined using liver tissue biopsies prior to drug treatment. As reported in previous studies, the repeated liver tissue biopsies before treatment have demonstrated their applicability to MASLD animal studies by suggesting that they can screen a validated MASLD model [12]. 

Though both TIC and CLO share the same pharmacological mechanism, many previous studies have reported that the effects and side effects differ between the two drugs. For instance, TIC has better anti-atherosclerotic activity than CLO because it can induce the expression of PON1 [21]. In addition, TIC downregulates the expression of oxidative stress-related genes (*prdx5*, *coq7*, and *Apd1*), while CLO does not; TIC also upregulates the expression of carboxylesterase 2B (*Ces2b*) genes, which are involved in lipid catabolism, more than CLO [21]. In addition to inhibiting the P2Y12 receptor, TIC inhibits equilibrative nucleoside transporter 1 [23,24], which transports adenosine in and out of cells. In patients with acute coronary atherosclerosis, TIC administration induces higher plasma adenosine concentrations than CLO [25]. Increased adenosine inhibits platelet aggregation, reduces inflammatory response, and is cardioprotective [23,26,27]. TIC also confers anti-inflammatory effects by enhancing adenosine-induced neutrophil migration [28]. These results are consistent with the present study and indicate that CLO and TIC have different effects. Unlike CLO, TIC significantly decreased steatosis and NAS. Similarly, the expression of de novo lipogenesis and ER stress markers was decreased by TIC significantly more than that by CLO. TIC decreased the expression of the markers of inflammation and decreased reactive oxygen species production, whereas CLO did not. In addition, using RNA sequencing data from a previous study, we confirmed that de novo lipogenesis gene expression was decreased more by TIC than by CLO and that treatment with TIC reduced the expression of lipid uptake genes (such as FABP1 and CD36) more than that with CLO. However, in patients who have other diseases concurrently with MASLD, the use of antiplatelet agents may not be beneficial [5]. Further studies are required to verify these potential limitations [5].

## 5. Conclusions

In conclusion, the present study suggested that TIC is more effective at reducing steatosis in MASLD than CLO. We confirmed that TIC treatment reduces the expression of de novo lipogenesis genes in animal experiments. In addition, TIC treatment reduced the expression of de novo lipogenesis genes and ER stress in HepG2 cells. However, since this mechanism has not yet been studied extensively, further experiments and studies are needed.

## Figures and Tables

**Table 1:** Sequences of primers.

Primer	Sequence
Mouse GAPDH	F: 5′-GTT GTC TCC TGC GAC TTC-3′
	R: 5′-GGT GGTCCA GGG TTT CTT-3′
Mouse SERPB1c	F: 5′-GAA ACA CTC AGC AGC CAC-3′
	R: 5′-CCA GCT TTG GAC CTG GGT-3′
Mouse FAS	F: 5′-CCC TTT TTG AGG AGG CCA AT-3′
	R: 5′-GCT TCA CGA CTC CAT CAC GA-3′
Mouse DATG2	F: 5′-ACT TCA CCT GGC TGG CAT TTG-3′
	R: 5′-GGT CAG CAG GTT GTG TGT CTT CA-3′
Mouse SCD1	F: 5′-AGA AGG GCG GAA AAC TGG AC-3′
	R: 5′-AGG CCG GGC TTG TAG TAC CT-3′
Mouse CHOP	F: 5′-CCA CCA CAC CTG AAA GCA GAA-3′
	R: 5′-AGG TGA AAG GCA GGG ACT CA-3′
Mouse Xbp1	F: 5′-TCA AAT GTC CTT CCC CAG AG-3′
	R: 5′-AAA GGG AGG CTG GTA AGG AA-3′
Mouse GRP78	F: 5′-CAT GGT TCT CAC TAA AAT GAA AGG-3′
	R: 5′-GCT GGT ACA GTA ACA ACT G-3′
Mouse MCP1	F: 5′-CCC AAT GAG TAG GCT GGA GA-3′
	R: 5′-TCT GGA CCC ATT CCT TCT TG-3′
Mouse TNF-α	F: 5′-CCG ATG GGT TGT ACC TTG TC-3′
	R: 5′-CGG ACT CCG CAA AGT CTA AG-3′
Mouse NOX2	F: 5′-CTG GTG TGG TTG GGG CTG AAT GTC-3′
	R: 5′-CAG AGC CAG TGC TGA CCC AAG GAG-3′
Mouse NOX4	F: 5′-CCG GAC AGT CCT GGC TTA TCT-3′
	R: 5′-TGC TTT TAT CCA ACA ATC TTC TTG TT-3′
Human β-actin	F: 5′-AGG AAG GAA GGC TGG AAG AG-3′
	R: 5′-AGA GCT ACG AGC TGC CTG AC-3′
Human SREBP1c	F: 5′-CTG CTG TCC ACA AAA GCA AA-3′
	R: 5′-CTC CAT GAG CAC GTC TGT GT-3′
Human FAS	F: 5′-ATA AGC CCT GTC CTC CAG GT-3′
	R: 5′-TGG AAG AAA AAT GGG CTT TG-3′
Human SCD1	F: 5′-GGC ATA ACA GCA GGA GC-3′
	R: 5′-CCA CAG CAT ATC GCA AG-3′
Human DGAT2	F: 5′-CTA CAG GTC ATC TCA GTG CT-3′
	R: 5′-GAA GTA GAG CAC AGC GAT GA-3′

**Table 2:** Information on primary antibodies.

Primary Antibody	Source (Company, Catalog Number)	Working Concentration
SREBP1c (A-4)	Santa Cruz Biotechnology, sc-365513	1:1000
FAS (B-10)	Santa Cruz Biotechnology, sc-8009	1:1000
SCD-1 (E-8)	Santa Cruz Biotechnology, sc-515844	1:1000
β-Actin (C-4)	Santa Cruz Biotechnology, sc-47778	1:1000

## Data Availability

Data are contained within the article.
